# Selection of a *Digitalis purpurea* Cell Line with Improved Bioconversion Capacity of Hydroquinone into Arbutin

**DOI:** 10.3390/life14010084

**Published:** 2024-01-04

**Authors:** Carmen Elena Pop, Ana Coste, Ana-Maria Vlase, Constantin Deliu, Mircea Tămaș, Tibor Casian, Laurian Vlase

**Affiliations:** 1 Department of Pharmaceutical Industry and Biotechnology, Faculty of Pharmacy, Iuliu Haţieganu University of Medicine and Pharmacy, 8 Victor Babeș Street, 400012 Cluj-Napoca, Romania; carmen.pop@umfcluj.ro; 2 Institute of Biological Research Cluj-Napoca, National Institute for Research and Development in Biological Sciences, 48 Republicii Street, 400015 Cluj-Napoca, Romania; deliu_konstantin@yahoo.com; 3 Department of Pharmaceutical Botany, Faculty of Pharmacy, Iuliu Haţieganu University of Medicine and Pharmacy, 8 Victor Babeș Street, 400012 Cluj-Napoca, Romania; mtbotanica@yahoo.com; 4 Department of Pharmaceutical Technology and Biopharmacy, Faculty of Pharmacy, Iuliu Haţieganu University of Medicine and Pharmacy, 8 Victor Babeș Street, 400012 Cluj-Napoca, Romania; casian.tibor@umfcluj.ro (T.C.); laurian.vlase@umfcluj.ro (L.V.)

**Keywords:** cell suspensions, *Digitalis purpurea* L., high yield arbutin production, hydroquinone

## Abstract

This study aimed to investigate the biotransformation capabilities of a hydroquinone-tolerant Digitalis purpurea cell line (DpHQ) for bioconverting hydroquinone (HQ) into arbutin, a compound with significant therapeutic and cosmetic applications. The research evaluated the influence of various HQ concentrations, feeding protocols, and carbon sources on arbutin bioconversion yield. By using HPLC-MS for the quantification of arbutin in biomass and medium, the study revealed that higher precursor (HQ) concentration led to a more pronounced growth inhibition under single dosing than sequential dosing. At lower sugar (3%) and precursor (4 mM HQ) levels, arbutin predominantly remained within the cells, whereas higher sugar (6%) and HQ (5–6 mM) levels promoted its release into the medium. Arbutin production ranged from 591 mg/L under single dosing to 3049 mg/L with sequential dosing, with the highest yield being achieved with 5 mM HQ in divided doses and 6% glucose. This study holds novelty for being the first to demonstrate the DpHQ’s tolerance to high concentrations of HQ and its efficient capabilities to bioconvert HQ to arbutin, indicating that D. purpurea is equipped with the enzymes required for this process. These aspects highlight its potential as a biotechnological source for arbutin synthesis.

## 1. Introduction

Arbutin is a compound of hydroquinone and D-glucose. The latter exists in α, β, or acyclic form in an aqueous solution, with β-anomer being a dominant form [1]. The stereoisomer β-arbutin is called arbutin, mainly found in plants, such as bearberry leaves (*Arctostaphylos uva-ursi* (L.) Spreng.) [2], pears [3], wheat, coffee, and tea [4].

In Romania, the primary natural arbutin sources are identified as *Arctostaphylos uva-ursi*, referenced in the European Pharmacopoeia 11th edition under the monograph ‘*Uvae ursi folium*’, and ‘*Vaccinium vitis-idaea*’. *Arctostaphylos uva-ursi*, a protected species, is found only in certain areas like the Apuseni Mountains in Alba County and the northern Oriental Carpathians in Suceava County [5]. This species was officially listed in the *Romanian Pharmacopoeia 7th edition* (1956–1965), but due to its protected status and restricted habitat, it is not a feasible option for extensive arbutin harvesting. To conserve the plant, since 1965, *Vitis-idaea folium*, also known as lingonberry leaves, has been used as a substitute [6]. The European Pharmacopoeia’s *Uvae ursi folium* monograph mandates a minimum arbutin content of 7.0%, in contrast to the *Romanian Pharmacopoeia*, which requires at least 3% arbutin in *Vitis-idaea folium* [7]. Consequently, the latter represents a comparatively less abundant source of this bioactive compound.

Arbutin is synthesized by attaching a glucose residue to one of hydroquinone’s phenolic hydroxyl groups. The glucose donor in this process is uridine diphosphate-glucose (UDP-glucose), and the transfer is catalyzed by an enzyme from the glucosyltransferase class named arbutin synthetase. This enzyme was first isolated from Rauwolfia serpentina cell suspensions [8,9]. Despite its low abundance, it was successfully detected, purified, and noted for its presence in this plant that naturally lacks arbutin. The enzyme exhibits low substrate specificity. The biotransformation process begins when hydroquinone is introduced into the culture medium, inducing the production of more UDP-glucose—a control mechanism demonstrated in Yokoyama et al.’s studies [10].

Arbutin was initially studied as a hydroquinone alternative for skin-lightening purposes [11]. Other beneficial properties attributed to the isomers include anti-oxidative [12], anti-microbial [13], anti-inflammatory [14], anticancer [15], and neuroprotective effects [16]. The potential applications of arbutin are growing, particularly in the pharmaceutical and cosmetic industries [1,4,17].

Given the substantial global and particularly Romanian market demand for natural supplements containing arbutin, the need to find alternative sources is imperative. This necessity is especially significant for conserving the protected species *Arctostaphylos uva-ursi*, which is vulnerable to risks associated with overharvesting and limited natural habitats. As a result, the growing demand for arbutin has driven research into finding efficient methods for its production and exploring its mechanism, effectiveness, and safety. Arbutin production mainly relies on plant extraction, chemical synthesis, microbial fermentation, and biotransformation [18]. Arbutin extraction from plants is hindered by its low or variable content depending on species, plant parts, developmental stages, and harvesting season [3,19,20]. Arbutin yield and purity also vary depending on the extraction and purification methods [20,21], while its chemical synthesis is also quite complicated and tedious [1,22]. Therefore, the biotechnological production of arbutin from hydroquinone under controlled conditions has been explored to meet the specific demands of the pharmaceutical and cosmetic industries [22]. An efficient alternative explored for arbutin production was via biotransformation in plant cell cultures. This approach exploits the glycosylation ability of plant cells to convert exogenous hydroquinone (HQ) to β-arbutin [23] and has the advantages of being independent of climate and other environmental factors, low energy consumption, reduced pollution, and high specificity [1]. HQ catalyzed β-arbutin synthesis was, thus, achieved in plant cell cultures of *Rauwolfia serpentina* [24], *Catharanthus roseus* cells [25], hairy roots of *Brugmansia candida* [26], *Capsicum annuum* [27], *Aronia melanocarpa* [22].

*Digitalis pupurea* L. (foxglove) is well known for its content in cardiac glycosides, including digitoxin and digoxin, with great therapeutic importance in the treatment of congestive heart failure [28]. Preliminary qualitative analysis performed in our laboratory has proven that *Digitalis purpurea* and *Digitalis lanata* plant cell suspensions are capable of biotransforming exogenously added hydroquinone into arbutin due to a non-specific enzyme glucosyl-transferase [29,30,31].

The aim of the present study was to examine the optimization of the biotransformation process by testing various dosages of the precursor (hydroquinone 4, 5, and 6 mM) and various methods of its administration (single versus sequential doses) in *Digitalis purpurea* cell suspension with high resistance to elevated hydroquinone concentrations.

## 2. Materials and Methods

### 2.1. Digitalis Purpurea Cell Cultures

The *D. purpurea* cell line used for the bioconversion of hydroquinone (DpHQ) was obtained through a multi-step selection process from a previously established F3 cell line [31]. The F3 cell line was initiated from seedlings resulting from in vitro germinated seeds collected from *D. purpurea* plants growing in the medicinal plants collection of “Alexandru Borza” Botanical Garden from Cluj-Napoca. A voucher specimen from which the seeds have been collected was deposited in the official Herbarium Collection of the Pharmaceutical Botany Department, Faculty of Pharmacy, “Iuliu Hațieganu” University of Medicine and Pharmacy from Cluj-Napoca, Romania, under the number 126.14.1.1.

After 6 successive subcultures at 21-day intervals on Murashige and Skoog (MS) [32], liquid medium supplemented with 1 mg/L of 2,4-dichlorophenoxyacetic acid (2,4-D) and 2 mg/L of 6-Benzylaminopurine (BA) [25], the F3 line was transferred for callus induction on the same nutrient medium solidified with agar (7.8 g/L) and supplemented with 1 mM hydroquinone. The resulting friable callus was subcultured 3 times at 21-day intervals on the above-mentioned solid medium devoid of hydroquinone. Subsequently, 2 g of friable callus was transferred to agitated MS liquid medium, supplemented with 0.2 g/L glutamine, 0.2 g/L hydrolyzed casein, 1.0 mg/L of 2,4-D, 2.0 mg/L of BA, and 3% sucrose as carbon source. The cell suspensions were subcultured at bi-weekly intervals over a two-month period, adjusting the inoculum-to-culture medium ratios at each subculturing stage (1:2, 1:3, 1:5, 1:10). These subcultures were conducted in 100 mL Erlenmeyer flasks containing 25 mL of culture medium. Periodic sieving through sterile nylon membrane filters of varying pore sizes (0.8 mm, 0.4 mm, and 180 µm) minimized cell aggregation and maintained the cultures in a dispersed state. For ensuing biotransformation experiments, 200 mL Erlenmeyer flasks with 50 mL of cell suspension and a 1:10 inoculum-to-culture medium ratio were employed. All cultures were agitated at 100 rpm on a rotary shaker and maintained at a temperature of 25 ± 1 °C under a 16 h photoperiod with a light intensity of 30 μmol/m^2^/sec.

### 2.2. Hydroquinone Feeding Experiments

Hydroquinone (HQ), purchased from Merck (Darmstadt, Germany), was solubilized in bi-distilled water and aseptically administered to the cell suspension cultures through a 0.22 μm Millipore membrane filter. Two distinct HQ feeding protocols, at concentrations of 5 mM and 6 mM, were implemented for the biotransformation studies. The first approach, based on Coste et al. [29], involved a single HQ administration on the 14th culture day, followed by sample collection at intervals of 3, 6, 12, 24, and 48 h for both arbutin production and growth profiling. The second strategy, adapted from Yokoyama and Inomata [10], implied a sequential HQ addition commencing on the 10th culture day. For the 5 mM concentration, 1 mM HQ was added initially on the 10th day, followed by 2 mM HQ on each subsequent day. To achieve the 6 mM concentration, 2 mM HQ was added daily for three consecutive days, beginning on the 10th day. Evaluation of arbutin yield from dry biomass and liquid medium, as well as growth rate assessment, was carried out on the 14th culture day. All hydroquinone-supplemented cultures were maintained under agitation at 100 rpm at a temperature of 25 ± 1 °C and a 16 h photoperiod, illuminated by cool white, fluorescent light at an intensity of 30 μmol/m^2^/sec.

### 2.3. Carbon Source Experiments

Sucrose and glucose were tested at two different concentrations, 3% and 6%, respectively. Carbon source concentration was increased through supplementation (a 3% increment) on the 10th day of culture.

### 2.4. Cell Growth

The growth rate of the control DpHQ cell line was determined by recording dry weight (DW) every 2 days, employing two distinct inoculum-to-culture medium ratios: 10% (*v*/*v*) and 20% (*v*/*v*). Growth profiles were also delineated for each biotransformation trial. For the first HQ feeding regimen, DW was ascertained at time intervals of 3, 6, 12, 24, and 48 h post-HQ application on the 14th day. In the case of the second HQ feeding scheme and varying carbon sources, DW was determined on day 14. Cells were separated from the liquid medium through vacuum filtration, as detailed by Evans et al. [33], and subsequently dried overnight at 80 °C. DW was recorded after drying.

### 2.5. Arbutin Extraction and Quantification

Arbutin was extracted and quantified following a methodology described by Pop et al. [34]. Specifically, 50 mg of dried powdered material was sonicated in 20 mL of methanol/water (5:95) solvent at 25 °C for 30 min. High-performance liquid chromatography–tandem mass spectrometry (HPLC-MS) was employed for arbutin quantification. The analysis utilized an Agilent 1100 Series HPLC system (Agilent Technologies, Inc., Santa Clara, CA, USA) equipped with a G1322A degasser, G1311A quaternary gradient pump, and a G1313A autosampler. The system was coupled with a mass spectrometer (Agilent Ion Trap 1100 SL from Agilent Technologies, Inc., Santa Clara, CA, USA). Chromatographic separation was achieved on a reversed-phase Zorbax SB-C18 column (100 mm × 3.0 mm i.d., 3.5 μm particle, Agilent Technologies, Inc., Santa Clara, CA, USA) at a set temperature of 45 °C. The mobile phase consisted of water with 50 μM sodium acetate at a flow rate of 1 mL/min and an injection volume of 5 μL. Arbutin detection employed single ion monitoring (SIM) mode in an ion trap mass spectrometer with positive ion electrospray ionization (ESI). Under these chromatographic conditions, arbutin exhibited a retention time of 1.6 min. The mass spectrometer’s parameters were set with a nebulizing gas (nitrogen) pressure of 60psi, a drying gas flow of 12 L/min, a drying gas temperature of 300 °C, and a capillary voltage of +4000 V. Chromatographic data processing was performed using ChemStation (vB01.03) and Data Analysis software (v5.3) from Agilent Technologies, Inc. (Santa Clara, CA, USA) [34]. Arbutin content was analyzed in both dry biomass and lyophilized culture media.

### 2.6. Experimental Design

The effect of independent variables on the investigated responses was evaluated through a Design of Experiments approach (DoE) (Table 1). The developed models were evaluated in terms of goodness of fit (R^2^), goodness of prediction (Q^2^), and validity by applying ANOVA. Surface contour plots were generated for interpretation purposes. The analysis of experimental data were performed using Modde 13.2.0 (Sartorius Stedim Biotech GmbH, Göttingen, Germany).

### 2.7. Statistical Analysis

Data were collected from three distinct experiments, with each experiment having three replicates. Within each replicate, four individual culture flasks were used to assess each experimental parameter and protocol. The experimental results were subjected to statistical analysis of variance (ANOVA) with Modde software, version 13.2.0 (Sartorius Stedim Biotech GmbH, Göttingen, Germany). *p* values below 0.05 were considered statistically significant.

## 3. Results

### 3.1. Growth Profile

**Figure 1:**
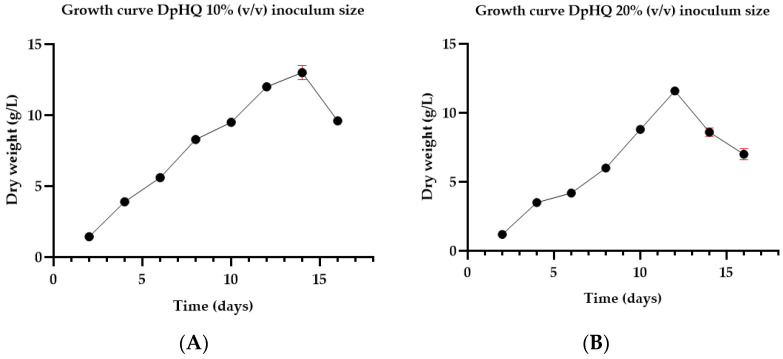
Digitalis purpurea DpHQ cell line growth profile (Dry weight—DW): (**A**) 10% (*v*/*v*) inoculum size (**B**) 20% (*v*/*v*) inoculum size. From 2 to 16—harvesting days. Data are presented as mean value ± SD (in red).

In order to estimate the optimum moment for hydroquinone administration, the growth rate of the DpHQ cell line was evaluated prior to biotransformation experiments at two different inoculum/culture medium ratios, at 10% (*v*/*v*) and 20% (*v*/*v*) inoculum size, respectively. Figure 1 depicts the growth profile, measured in terms of dry weight (DW), of the DpHQ cell line under two different inoculum sizes: 10% (*v*/*v*) and 20% (*v*/*v*). Data were recorded at various harvesting days ranging from day 2 to day 16.

The growth rate for the 10% (*v*/*v*) inoculum size showed a marked increase in dry weight from day 6 onwards, reaching a maximum increase on day 14 (exponential phase), representing about a nine-fold increase (13 g/L) over the initial dry weight. Starting with the 14th day of culture, a gradual reduction in cell density was observed (Figure 1A). For the 20% (*v*/*v*) inoculum size, the dry matter showed a three-times increment between the 2nd and 4th days of culture. After a two-day stationary period between the 4th and 6th days of culture, the biomass quantity increased at a sustained rate until the 12th day of culture, reaching its maximum development (11.6 g/L of DW). Starting from the 12th day of culture, the suspension began to enter the declining phase of its development (Figure 1B). Biomass productivity was inversely proportional to the size of the initial inoculum, with maximum growth attained on the 14th day of culture when using an inoculum density of 10% (*v*/*v*).

### 3.2. Feeding Protocols Outcomes

Cell growth varied considerably, depending on hydroquinone and carbon source concentration (Table 2 and Table 3). In single-dose HQ feeding protocol 1, a more significant biomass growth inhibition was recorded with increased hydroquinone concentrations (Table 2) compared to feeding protocol 2, where hydroquinone was added in sequential doses (Table 3). It is important to underline here that the timing of HQ administration was different (day 14 for protocol 1, day 10 for protocol 2); therefore, initial biomass levels at HQ feeding moment were different, 13 g/L and 9.5 g/L, respectively. In HQ feeding protocol 2, supplementation with higher sucrose or glucose concentrations (6%) provided a higher protective effect against the damaging effect of HQ on dry weight accumulation (Table 3). However, when observing the dry weight, high sucrose concentrations still stood out compared to glucose (Table 3). Overall, the *D. purpurea* DpHQ cell line cultured in the present study was able to tolerate a high dosage of hydroquinone, revealing only slight differences between dry weight accumulation at low and high precursor concentrations. Based on these preliminary data and to avoid damaging the cells, it was considered that the optimal inoculum size for the biotransformation experiments is represented by the 10% (*v*/*v*) and that the best starting point for hydroquinone administration would be starting with day 10 until the 14th day of culture. Therefore, it was decided to test two HQ feeding protocols: (1) one dose feeding on day 14 (exponential phase) and arbutin quantification at 3, 6, 12, 24, and 48 h; (2) gradual HQ feeding starting with day 10 until day 13 and arbutin quantification starting with day 14 for HQ feeding protocol 2.

### 3.3. Biotransformation Experiments Results

**Figure 2:**
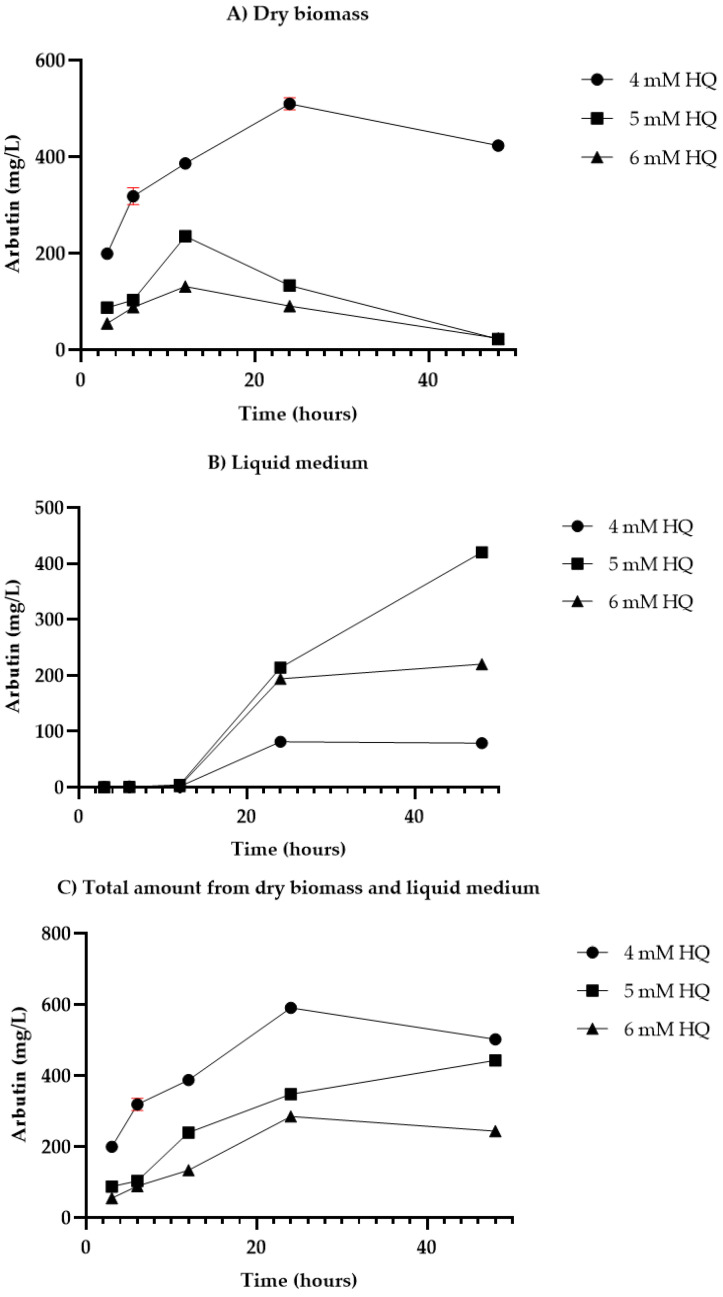
Arbutin yield (mg/L) in *D. purpurea* plant cell suspensions cultured on MS liquid medium supplemented with 0.2 g/L glutamine, 0.2 g/L hydrolyzed casein, 1.0 mg/L 2,4-D and 2.0 mg/L BAP, pH = 5.7, HQ feeding protocol 1—single dose (4, 5, and 6 mM HQ): (**A**) arbutin content in dry biomass; (**B**) arbutin content in liquid medium; (**C**) total amount of arbutin (dry biomass+liquid medium). Data are presented as mean value ± SD (in red).

Figure 2 provides insights into the yield of arbutin (mg/L) produced in *D. purpurea* plant cell suspensions cultured under specific conditions. It covers three subcomponents: (A) arbutin content in dry biomass, (B) arbutin content in liquid medium, and (C) the total amount of arbutin combining both dry biomass and liquid medium. The data are generated from experiments applying the first HQ feeding protocol with single doses of 4, 5, and 6 mM HQ.

For both HQ feeding protocols, arbutin was detected in the extracts from dry biomass as well as in the samples from the culture medium. Within 3–12 h following HQ single-dose administration, DpHQ cell cultures retained most of the arbutin (55.88–306.4 mg/L) in the cells, releasing only trace amounts of arbutin in liquid medium (0.2–4.5 mg/L) (Figure 2A,B). The highest levels of arbutin were registered in both biomass and liquid medium after 24 and 48 h of HQ undivided dose administration. At lower HQ concentrations (4 mM), most of the arbutin (509.6 mg/L) accumulated within the cells after 24 h of HQ addition (Figure 2A). The overall arbutin content (cells + liquid medium) decreased with HQ concentration, reaching the highest total amount of arbutin (591.2 mg/L) at 24 h of 4 mM HQ single dose administration (Figure 2C). Increased precursor doses (5–6 mM) stimulated the release of arbutin into the culture medium with more significant amounts (220–420 mg/L) at 48 h after precursor administration.

For HQ sequential feeding protocol 2, the outcomes varied depending on HQ concentration and carbon source. Thus, at lower carbon sources (3%) and precursor concentrations (4 mM), most of the arbutin was retained in the cells, while at higher sugar (6%) and HQ (5–6 mM) concentrations, the cells began to release the arbutin in the culture medium (Table 4). Overall, higher efficiency of the biotransformation process was observed for 3% sucrose with divided precursor administration compared to HQ single-dose feeding, especially at higher HQ concentrations (Table 4). Thus, at 3% sucrose, arbutin production increased eight times (762:88 mg/L) for 5 mM HQ and 17 times (955:55 mg/L) for 6 mM HQ sequential administration compared to arbutin levels at 3% sucrose and at 3 h after HQ single dose administration (Figure 2C and Table 4, respectively). However, compared to arbutin quantification at 48 h in protocol 1, the increment is about two-fold for a 5 mM precursor dose (762:443 mg/L) and four-fold for a 6 mM HQ dose (955:244 mg/L), respectively (Figure 2C and Table 4, respectively). The highest arbutin levels for HQ feeding protocol 2 (Table 4) were registered at 5 mM hydroquinone administration and high carbon source concentrations, reaching 2665 mg/L for 6% sucrose and a maximum of 3049 mg/L for 6% glucose concentration (Table 4). Higher carbon source concentrations (6%) stimulated arbutin release in the culture medium, indifferent to the concentration of hydroquinone (Table 4). In response to various sucrose concentrations, cell cultures with 6% sucrose not only produced the highest dry biomass but also high quantities of arbutin. For HQ sequential feeding protocol 2, the 6% glucose concentration induced the highest yield of arbutin, while 3% glucose revealed the lowest amounts of arbutin (Table 4). Comparing the content of arbutin in both administration experiments (single dose, divided doses), it was noticed that regardless of the precursor concentration, arbutin yield was stimulated when hydroquinone was added in divided doses (Figure 2C and Table 4, respectively).

### 3.4. Outcomes of Fitting the Data with the Models

Table 5 provides the performance of the evaluated experimental design, focusing on two essential statistical measures: R^2^, the Coefficient of Determination, and Q^2^, indicative of the predictive ability. R^2^ reflects the percentage of response variance that our model explains, with higher values indicating a better fit to the experimental data. This is particularly relevant for assessing the model’s capacity to elucidate variations in key responses such as cell growth, dry biomass, and arbutin content [35]. The Q^2^ value, on the other hand, assesses the model’s ability to predict new, unseen data, a critical measure of its reliability and applicability. Like R^2^, a higher Q^2^ value suggests better predictive ability as it is an indicator of how good the model is expected to perform on external or validation datasets. Q^2^ should be close to R^2^ (<0.3 difference between them), indicating that the model not only fits the experimental data well but also has good predictive power [36].

As indicated by the results given in Table 5, significant models (*p* < 0.05) were developed for all responses because there is a statistically significant difference between the percentage of variation that can be explained and the percentage of variation that cannot be modeled. Thus, it is possible to model the variation of the responses by the studied factors.

**Figure 3:**
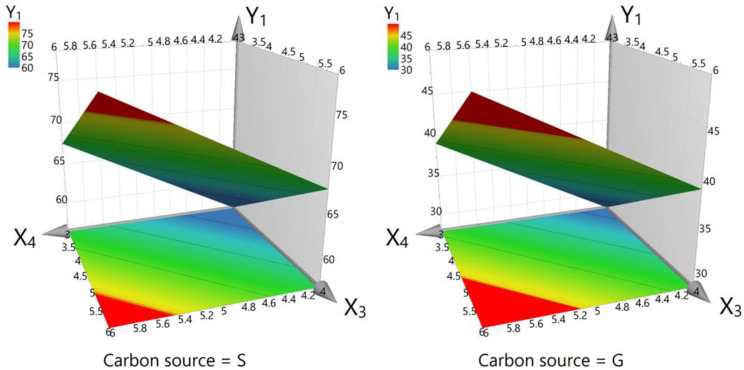
Surface contour plot for cell growth (factor Y_1_). X_3_—carbon source level (3% or 6%) and X_4_—hydroquinone concentration (4 mM, 5 mM, or 6 mM). S—sucrose, G—glucose. The red zones highlight the optimal working conditions for attaining maximum cell growth.

**Figure 4:**
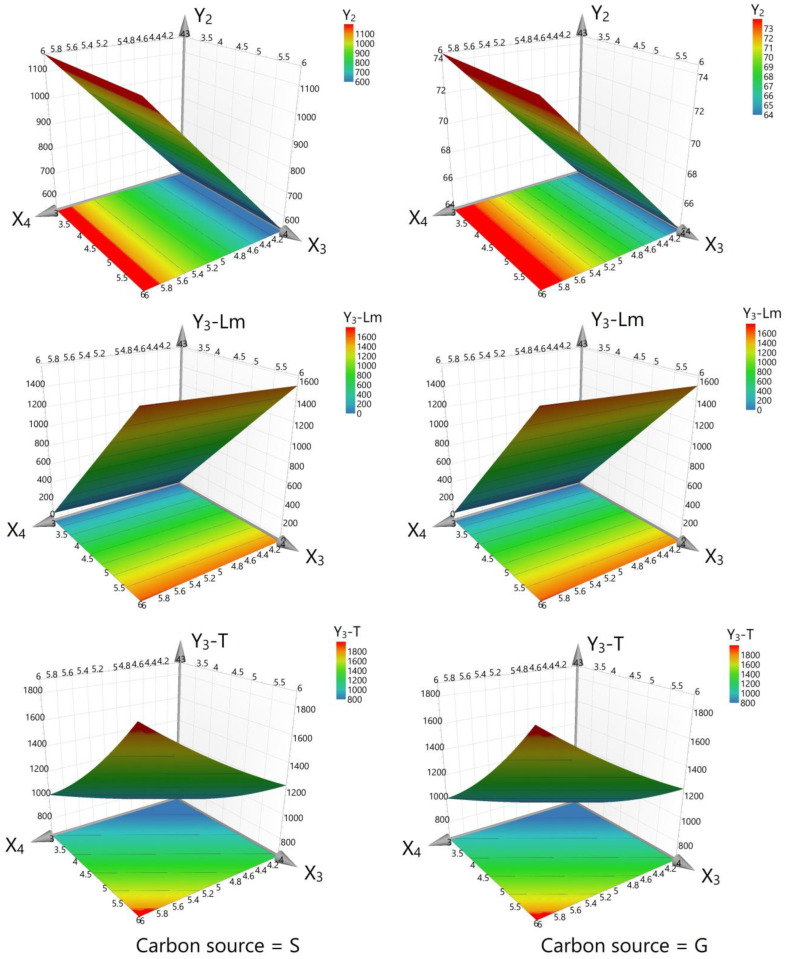
Surface contour plot for dry biomass (factor Y_2_). Y_3_-Lm—Arbutin content in liquid medium (Lm) and Y_3_-T—Total amount of arbutin (after applying protocol 2). S—sucrose, G—glucose. The red zones show the optimal working conditions for obtaining maximum arbutin production.

**Figure 5:**
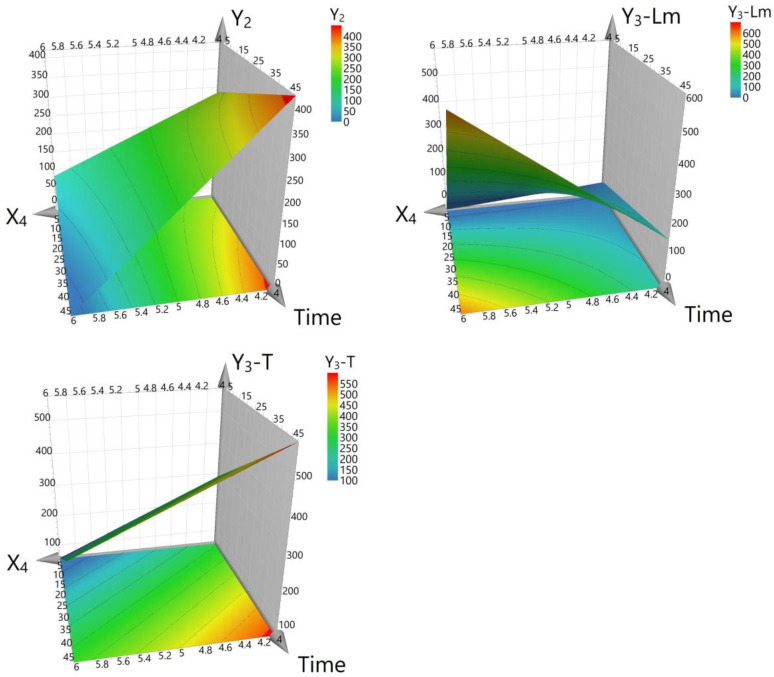
The influence of hydroquinone concentration (factor X_4_) and its adding time on dry biomass (response Y_2_), arbutin content in liquid medium (response Y_3_-Lm), and total amount of arbutin (response Y_3_-T). The red zones indicate the optimal working conditions for achieving maximum arbutin production.

In interpreting the results of this experiment, the interplay between the variables and their individual and combined impacts on cell growth, dry biomass, arbutin content from liquid medium, and total arbutin production must be considered. The results offer a nuanced view of these relationships, as depicted in the corresponding figures (Figure 3, Figure 4 and Figure 5). The graphics illustrate that the working parameters falling within the red zones are optimal for achieving the maximum yield for the investigated responses (cell growth, dry biomass, and arbutin content).

## 4. Discussion

### 4.1. Growth Profile

It has been shown that inoculum size (density) and age significantly influence biomass growth in cell cultures of different plant species. Most studies reported increased biomass productivity with inoculum size [37,38,39], while other studies showed that cultures with higher initial inoculums seemed to show limited growth when compared with cultures with lower initial inoculums [40,41,42]. In a previous experiment, the *Digitalis lanata* plant cell cultures with lower inoculum size proved to grow faster in the first days after the inoculation than variants with higher amounts of inoculum [30]. This is likely because higher inoculum sizes might cause nutrient oxygen depletion in the culture medium [42]. A better growth is justified at certain inoculum densities depending on plant species, culture conditions, as well as on the specific surface area of the cells and the dispersed nature of cell suspensions, which can make them more prone to leakage of key growth factors/cellular contents to medium [37]. However, similar inoculum/culture medium ratios (10–12.5% *v*/*v*) were reported as optimum in the related *Digitalis lanata* species [43,44].

Cell growth varied considerably, depending on hydroquinone and carbon source concentration (Table 2 and Table 3). Similar results were reported by Dantas et al. [45] in *Hancornia speciosa* cell suspensions, where sucrose favored greater biomass accumulation. In the current experiment, the highest biomass was recorded at a high sucrose concentration (6%) for all HQ doses (Table 3). Several studies showed that disaccharides, especially sucrose, are favorable for biomass accumulation when applied in higher concentrations than the standard dosage of 30 g/L. See et al. [46] reported significantly higher dried cell weight in plant cell suspensions of *Melastoma malabathricum* when the culture medium was supplemented with 45 g/L sucrose, while a decreasing trend was registered when the sucrose level was further increased at 60 g/L or higher. Increased dry weight values were also reported in cell suspensions of *Jatropha curcas* and *Salvia leriifolia* with 40% sucrose [47,48].

Prior research, notably by Yokoyama et al., underscored the critical role of carbon source choice and concentration in arbutin production. In *Catharanthus roseus* cell cultures, a 6% sucrose supplement significantly increased arbutin yield by two to three times, up to 1.5–2 g/L [49]. Yokoyama’s further studies revealed that mature cells with larger vacuoles are more efficient in hydroquinone biotransformation, although this capacity is limited in batch systems due to nutrient depletion [50]. Notably, a 9.2 g/L arbutin production was achieved in a 20 L fermenter, suggesting that biotransformation could surpass chemical synthesis due to its high conversion rate and lack of secondary reactions [10]. Drawing on these findings, our study also examined the influence of different sugars on arbutin production, highlighting sucrose as a particularly effective carbon source, leading to higher arbutin yields.

The damaging effect of high dosages of hydroquinone on plant cells cultured in vitro is a well-known phenomenon [10,51]. However, the *D. purpurea* DpHQ cell line cultured in the present study was able to tolerate a high dosage of hydroquinone, revealing only slight differences between dry weight accumulation at low and high precursor concentrations. This indicates the stability of the suspension against the toxic effect of hydroquinone, which might be due to the preculturing of the callus used for DpHQ cell suspension initiation on HQ feed MS medium.

### 4.2. Biotransformation Experiments

The agitated *D. purpurea* DpHQ cell line was able to effectively convert the supplied hydroquinone into arbutin. High HQ doses (5–6 mM) stimulated the release of arbutin into the culture medium at 48 h after precursor administration. Similar results were reported in *Datura innoxia* plant cell suspensions where no arbutin remained in the cells at high HQ concentrations [51]. This might be explained by the longer exposure of plant cells to the toxic effect of hydroquinone, which led to cell disruption and arbutin release into the culture medium.

For HQ sequential feeding protocol 2, at lower carbon source and precursor concentrations, most of the arbutin was retained in the cells, while at higher concentrations, the cells began to release the arbutin in the culture medium. Similar results were reported in *Vigna radiata* and *Echinacea purpurea* cell cultures where lower HQ concentrations (0.5–1.8 mM) stimulated arbutin accumulation mainly in cells and only in insignificant amounts in culture medium [52]. However, in our experiments, a higher efficiency of the biotransformation process was observed for 3% sucrose with divided precursor administration compared to HQ single-dose feeding, especially at higher HQ concentrations. Thus, we found that higher carbon source levels (6%) stimulated not only dry biomass yield but also arbutin release into the culture medium, indifferent to hydroquinone concentration. Sucrose was reported to act as an external energy source that stimulates the absorption of mineral nutrients present in the culture medium, stimulating the production of the energy required for metabolite synthesis [53,54,55]. Moreover, many studies have pointed out that yields of in vitro cell cultures for secondary metabolite biosynthesis were highly dependent on the type and concentration of carbohydrates used in the medium [56,57]. Similar results were reported in other types of in vitro cultures. Thus, sucrose or glucose at all tested concentrations (30–120 g/L sugars compared to sugar-free medium) enhanced arbutin production from 8- to 12-fold in agitated shoot cultures of *Aronia melanocarpa* [22]. However, in our experiments, for HQ sequential feeding protocol 2, the 6% glucose concentration induced the highest yield of arbutin, while 3% glucose revealed the lowest amounts of arbutin. Arbutin yield in both administration experiments (single dose, divided doses), regardless of the precursor concentration, was stimulated when hydroquinone was added in divided doses. Similar results were reported in *Datura innoxia* cell suspension cultures, where the repeated addition of lower HQ concentrations increased arbutin yield and cell density [51]. Higher arbutin levels up to 9.2 g/L (45% of cell dry weight) were also reported in *Catharanthus roseus* cell cultures at lower precursor concentrations (4 mM HQ) and sequential addition [25]. Other studies reported increased arbutin production rates (18 g/L) in *Rauwolfia serpentina* high-density cell suspensions at increased culture volumes (1.5 L working volume) [24]. Higher hydroquinone concentrations (15.57 mM) induced lower arbutin production rates in cell suspension cultures of *Capsicum annuum* (0.3 g/L), *Datura fastuosa* (0.015 g/L), and *Solanum aculeatissimum* (0.071 g/L) grown in MS medium with 2% sucrose, 2.4-D, and BAP and fed 5 days before harvest [58]. However, even at lower HQ concentrations (0.5–1.8 mM), the arbutin content ranged from 0.78 to 1.89% and 2.00 to 3.55% of dry weight in *Vigna radiata* and *Echinacea purpurea* plant cell suspensions [52].

HQ-catalyzed β-arbutin synthesis was also reported in other types of in vitro plant cultures. Thus, higher arbutin levels (8.27 g/100 g dry weight) were observed in agitated shoot cultures of *Aronia melanocarpa* following the HQ addition in two portions, however, in lower concentration (3.5 mM) [22]. High bioconversion rates were reached in hairy roots of *Brugmansia candida* at low hydroquinone concentrations (0.18–0.36 mM) and 30–120 g/L sugars (sucrose, glucose, mannitol, and sorbitol) supplementation, with sucrose and glucose as most effective in arbutin yield improvement [26]. After one week of incubation with hydroquinone, the percentage of arbutin was 5.08% in callus cultures of *Schisandra chinensis*, with the substance being released into the culture medium [59]. Yan et al. [60] reported 2.2 g/L arbutin production in transformed root cultures of *Polygonum multiflorum*, with a bioconversion rate of 81.45% at 72 h after 10 mM HQ addition. In these cultures, arbutin was identified in both the biotransformation medium and the dry biomass. Arbutin yields ranging from 0.17% to 3.1% were acquired in *Digitalis lanata* plant cell suspensions with arbutin accumulation exclusively in cells (dry biomass) [30]. The biotransformation capacity of *Digitalis purpurea* plant cell suspensions was proved only by qualitative thin-layer chromatography (TLC) analysis by using lower HQ concentrations (1 mM) [31].

The overall content of arbutin (3.05 g/L) obtained in *D. purpurea* cell suspensions is lower than the contents reported in plant cell cultures of some plant species, as well as compared to the 11th edition of the European Pharmacopoeia, which reports a 7.0% arbutin content for bearberry leaf (*Uvae ursi folium*) [7]. This might be explained by the different bioconversion capacities of the cells in different plant species and by the particularities of culture conditions (precursor concentration, cell density, culture volume, carbon source, aeration, etc.). Hence, the obtained results are interesting from a practical perspective.

To the best of our knowledge, this is the first report on the optimization process of hydroquinone bioconversion to arbutin in *D. purpurea* plant cell suspensions. However, further optimization of the biotransformation process is required for *D. purpurea* cell cultures to be considered as a potential biotechnological source of arbutin. Controlling parameters such as HQ addition time, concentration, culture volume, cell density, and sugar concentration have a significant influence on β-arbutin production. Currently, β-arbutin synthesis by plant cell culture method still cannot meet the requirements for industrial-scale production. However, these experiments clearly demonstrate the capacity of *D. purpurea* plant cell cultures to produce arbutin in higher amounts than other plant species.

### 4.3. Interpretation of the Experimental Design Results after Fitting the Data with the Models

In the context of optimizing the feeding protocol for selecting a *D. purpurea* cell line with an enhanced bioconversion capacity of HQ into arbutin, the current experimental data present a multifaceted understanding of the interactions between various factors and their collective impact on cell line selection criteria, including cell growth, biomass accumulation, and arbutin synthesis.

Our findings suggest that neither the concentration of HQ nor the carbon source level alone significantly affected cell growth, with *p*-values of 0.146 and 0.093, respectively (Figure 3). This result suggests that these factors, when varied independently, do not affect the growth of the cells within the experimental setup and, therefore, are not critical to cell growth under the specific conditions tested. However, the type of carbon source plays a critical role, with sucrose showing a positive effect (*p* = 0.001) on growth, which is consistent with higher-than-average outcomes when used in the experiments. In contrast, glucose (G) is negatively correlated with growth (*p* = 0.001), implying that it may be detrimental to cell health under the given conditions.

Dry biomass accumulation, an indicator of cell line growth, was positively affected by HQ and sucrose with *p*-values of 0.002 and <0.001, respectively, and negatively by glucose (*p* < 0.001), as shown in Figure 4. Furthermore, an interaction effect (*p* = 0.002) was observed, wherein the presence of sucrose augmented the positive impact of HQ on dry biomass production, a desirable trait for cell lines tasked with bioconversion. However, when glucose was used, the facilitative effect of HQ on biomass was diminished, suggesting that sucrose is a more favorable carbon source for optimizing HQ bioconversion efficiency in the *D. purpurea* cell line.

In the analyses of the arbutin content from the liquid medium (Y_3_-Lm), only the carbon source concentration showed a significant positive effect (*p* < 0.001), which is essential for promoting an environment suitable for bioconversion. The concentration of HQ did not significantly change the arbutin content in the liquid medium (*p* = 0.828), suggesting it does not play a decisive role in the selection of the cell line for this parameter.

Total arbutin production, the final point of the bioconversion process, demonstrated a nuanced response. While HQ concentration alone did not significantly impact arbutin production (*p* = 0.110), carbon source concentration increased arbutin yield (*p* = 0.011), and the HQ adding time significantly enhanced it (*p* < 0.001), as per Figure 5. A notable decrease in arbutin production with higher HQ concentrations (*p* < 0.001) suggests a complex bioconversion dynamic that warrants further investigation to optimize the feeding protocol.

The interaction between HQ concentration, adding time and sequence, and their effects on biomass, as well as arbutin production, further elucidates the optimal conditions for selecting a *D. purpurea* cell line with superior bioconversion capacity. An increase in HQ concentration corresponded with a decreased biomass yield (*p* < 0.001), and although the adding time of HQ did not significantly influence the biomass (*p* = 0.868), it had a positive impact on the bioconversion as reflected in outcomes obtained in liquid medium and overall arbutin production.

These results highlight the significance of carbon source selection, particularly sucrose, and the timing of hydroquinone feeding in the bioconversion process. The data point to the potential for optimized feeding protocols that enhance the bioconversion efficiency of *D. purpurea* cell lines, facilitating improved arbutin production.

## 5. Conclusions

The present study underscores the importance of cell proliferation and hydroquinone (HQ) dosing strategies in the biotransformation process to arbutin within *Digitalis purpurea* cell cultures (DpHQ). The main findings demonstrate the substantial impact of HQ concentration and dosing methods (single vs. sequential) on both cell growth and the yield of arbutin production. Furthermore, the research highlights that the concentration of the carbon source, along with the HQ precursor, is crucial in determining whether arbutin is predominantly retained within the cells or released into the liquid medium. Optimal conditions for maximizing arbutin production were identified, with higher concentrations of sucrose and glucose being particularly favorable to facilitating arbutin release into the medium.

Although this research was conducted on a pilot scale, it lays the groundwork for the potential upscale to industrial levels. The culture conditions and media used in this study are not only cost-effective but also adaptable for use in large-scale bioreactors, making this approach a viable option for the commercial production of arbutin. While further optimization remains imperative for establishing *D. purpurea* cell cultures as a viable biotechnological source of arbutin, our findings highlight the comparative advantage of *D. purpurea* in natural product synthesis over other plant species.

## Figures and Tables

**Table 1:** Independent and dependent variables of experimental design evaluated for DpHQ cell line for bioconversion of hidroquinone to arbutin.

Variables	Levels			
Independent Variables (Factors)				
Feeding protocol (X_1_)	1—single administration		2—sequential administration	
Carbon source (X_2_)	Sucrose (S)		Glucose (G)	
Carbon source level (X_3_)	3%	6%	3%	6%
Hidroquinone concentration (X_4_)	4 mM	5 mM	6 mM	
Dependent variables (responses)				
Cell growth (Y_1_)				
Dry biomass (dry weight) (Y_2_) (g/L)				
Arbutin content (Y_3_) (mg/L)	Dry biomass (Y_3_-Dm)			
	Liquid medium (Y_3_-Lm)			
	Total amount (Y_3_-T)			

**Table 2:** Dry biomass yields from hydroquinone feeding protocol 1—Biotransformation studies using 3% sucrose as the experimental feeding protocol.

Time (hours)	Dry Biomass (g/L)			
	Control *	4 mM HQ **	5 mM HQ	6 mM HQ
3	13.00 ± 1.00	12.00 ± 0.26	11.12 ± 0.15	10.84 ± 0.45 ^#^
6	12.00 ± 0.36	11.38 ± 0.41	10.76 ± 0.12	10.56 ± 0.15
12	11.40 ± 0.38	11.06 ± 0.01	10.28 ± 0.32	10.25 ± 0.12
24	10.40 ± 0.17	9.81 ± 0.16	9.74 ± 0.21	9.39 ± 0.19 ^#^
48	9.60 ± 0.20	7.99 ± 0.19 ^#^	7.87 ± 0.26 ^#^	7.23 ± 0.11 ^#^

* Control—no treatment; ** HQ—hidroquinone. ^#^—statistically significant versus control (*p* < 0.05).

**Table 3:** Dry biomass yields from hydroquinone sequential feeding protocol 2—Biotransformation studies using 3% and 6% sucrose, 3% and 6% glucose, respectively, as the experimental feeding protocol.

Carbon Source	Dry Biomass (g/L)			
	Hidroquinone Concentration			Control (No Treatment)
	4 mM	5 mM	6 mM	
3% sucrose	8.00 ± 0.60 ^#^	7.50 ± 0.10 ^#^	7.00 ± 0.78 ^#^	9.50 ± 0.30
6% sucrose	9.00 ± 0.66	8.60 ± 0.20	8.00 ± 0.56	
3% glucose	7.80 ± 0.54 ^#^	7.30 ± 0.36 ^#^	7.00 ± 0.30 ^#^	
6% glucose	8.20 ± 0.30 ^#^	7.80 ± 0.18 ^#^	7.10 ± 0.20 ^#^	

^#^—statistically significant versus control (*p* < 0.05).

**Table 4:** Arbutin yield in DpHQ cell line cultured under varying concentrations of sucrose and glucose and sequential hidroquinone feeding according to protocol 2.

	**Arbutin amount (mg/L)**					
	4 mM HQ *		**5 mM HQ**		**6 mM HQ**	
**Sucrose**	**3%**	**6%**	**3%**	**6%**	**3%**	**6%**
Dry biomass	498.40 ± 2.26	611.50 ± 2.46	761.60 ± 11.13	878.06 ± 3.63	952.45 ± 4.62	1004.20 ± 3.51
Liquid medium	0.23 ± 0.05	1049.56 ± 3.03	0.86 ± 0.06	1786.99 ± 9.31	2.77 ± 0.13	1356.33 ± 14.74
Total	498.63 ± 2.30	1661.06 ± 5.46	762.46 ± 11.07	2665.05 ± 5.73	955.22 ± 4.67	2360.53 ± 11.74
Conversion rate	45.827%	96.46%	56.66%	97.82%	58.53%	96.8%
	**Arbutin amount (mg/L)**					
	**4 mM HQ**		**5 mM HQ**		**6 mM HQ**	
**Glucose**	**3%**	**6%**	**3%**	**6%**	**3%**	**6%**
Dry biomass	23.10 ± 1.15	110.24 ± 3.39	42.00 ± 2.00	84.95 ± 2.05	77.00 ± 2.65	77.70 ±1.73
Liquid medium	0.30 ± 0.03	1125.80 ± 2.11	0.34 ± 0.06	2964.25 ± 4.68	0.00 ± 0.00	1537.10 ± 14.08
Total	23.40 ± 1.18	1236.04 ± 5.37	42.34 ± 2.04	3049.20 ± 4.68	77.00 ± 2.65	1650.80 ± 12.90
Conversion rate	9.46%	94.8%	17.12%	98.89%	31.13%	97.6%

* HQ—hidroquinone.

**Table 5:** Summary of fit.

Parameter	Cell Growth	Arbutin Content					
		Dry Biomass		Liquid Medium		Total Amount	
		FP 1 *	FP 2 **	FP 1	FP 2	FP 1	FP 2
R^2^	0.794	0.689	0.964	0.876	0.725	0.787	0.591
Q^2^	0.670	0.408	0.880	0.548	0.665	0.656	0.423
*p* (ANOVA)	0.004 ^#^	0.0039 ^#^	<0.001 ^#^	<0.001 ^#^	0.0029 ^#^	<0.001 ^#^	0.0177 ^#^

* FP 1—feeding protocol 1; ** FP 2—feeding protocol 2; ^#^ *p* < 0.05—statistically significant.

## Data Availability

The data presented in this study are available in the article.

## References

[1] Boo Y.C. (2021). Arbutin as a Skin Depigmenting Agent with Antimelanogenic and Antioxidant Properties. Antioxidants.

[2] Asensio E., Vitales D., Pérez I., Peralba L., Viruel J., Montaner C., Vallès J., Garnatje T., Sales E. (2020). Phenolic Compounds Content and Genetic Diversity at Population Level across the Natural Distribution Range of Bearberry (*Arctostaphylos uva-ursi*, Ericaceae) in the Iberian Peninsula. Plants.

[3] Cui T., Nakamura K., Ma L., Li J.-Z., Kayahara H. (2005). Analyses of Arbutin and Chlorogenic Acid, the Major Phenolic Constituents in Oriental Pear. J. Agric. Food Chem..

[4] Migas P., Krauze-Baranowska M. (2015). The Significance of Arbutin and Its Derivatives in Therapy and Cosmetics. Phytochem. Lett..

[5] Sârbu I., Ştefan N., Oprea A., Victor B. (2013). Plante Vasculare din Romania: Determinator Ilustrat de Teren.

[6] (2010). FARMACOPEEA Romana.

[7] European Directorate for the Quality of Medicines (2022). European Pharmacopoeia.

[8] Hefner T. (2002). Arbutin Synthase, a Novel Member of the NRD1β Glycosyltransferase Family, Is a Unique Multifunctional Enzyme Converting Various Natural Products and Xenobiotics. Bioorg. Med. Chem..

[9] Hefner T., Stöckigt J. (2003). Probing Suggested Catalytic Domains of Glycosyltransferases by Site-directed Mutagenesis. Eur. J. Biochem..

[10] Yokoyama M., Inomata S., Bajaj Y.P.S. (1998). Catharanthus roseus (Periwinkle): In Vitro Culture, and High-Level Production of Arbutin by Biotransformation. Medicinal and Aromatic Plants X.

[11] Sugimoto K., Nishimura T., Nomura K., Sugimoto K., Kuriki T. (2004). Inhibitory Effects of α-Arbutin on Melanin Synthesis in Cultured Human Melanoma Cells and a Three-Dimensional Human Skin Model. Biol. Pharm. Bull..

[12] Seyfizadeh N., Tazehkand M.Q., Palideh A., Maroufi N.F., Hassanzadeh D., Rahmati-Yamchi M., Elahimanesh F., Borzoueisileh S. (2019). Is Arbutin an Effective Antioxidant for the Discount of Oxidative and Nitrosative Stress in Hep-G2 Cells Exposed to Tert-Butyl Hydroperoxide?. Bratisl. Med. J..

[13] Jurica K., Gobin I., Kremer D., Čepo D.V., Grubešić R.J., Karačonji I.B., Kosalec I. (2017). Arbutin and Its Metabolite Hydroquinone as the Main Factors in the Antimicrobial Effect of Strawberry Tree (*Arbutus unedo* L.) Leaves. J. Herb. Med..

[14] Lee H.-J., Kim K.-W. (2012). Anti-Inflammatory Effects of Arbutin in Lipopolysaccharide-Stimulated BV2 Microglial Cells. Inflamm. Res..

[15] Zeng X., Liu H., Huang Z., Dong P., Chen X. (2022). Anticancer Effect of Arbutin on Diethylnitrosamine-Induced Liver Carcinoma in Rats via the GRP and GADD Pathway. J. Environ. Pathol. Toxicol. Oncol..

[16] Zhao J., Kumar M., Sharma J., Yuan Z. (2021). Arbutin Effectively Ameliorates the Symptoms of Parkinson’s Disease: The Role of Adenosine Receptors and Cyclic Adenosine Monophosphate. Neural. Regen. Res..

[17] Saeedi M., Khezri K., Seyed Zakaryaei A., Mohammadamini H. (2021). A Comprehensive Review of the Therapeutic Potential of A-arbutin. Phytother. Res..

[18] Xu W.-H., Liang Q., Zhang Y.-J., Zhao P. (2015). Naturally Occurring Arbutin Derivatives and Their Bioactivities. Chem. Biodivers..

[19] Tůmová L., Dolečková I., Hendrychová H., Kašparová M. (2017). Arbutin Content and Tyrosinase Activity of Bergenia Extracts. Nat. Prod. Commun..

[20] Sasaki C., Ichitani M., Kunimoto K.-K., Asada C., Nakamura Y. (2014). Extraction of Arbutin and Its Comparative Content in Branches, Leaves, Stems, and Fruits of Japanese Pear *Pyrus pyrifolia* cv. Kousui. Biosci. Biotechnol. Biochem..

[21] Cho J.-Y., Park K.Y., Lee K.H., Lee H.J., Lee S.-H., Cho J.A., Kim W.-S., Shin S.-C., Park K.-H., Moon J.-H. (2011). Recovery of Arbutin in High Purity from Fruit Peels of Pear (*Pyrus pyrifolia* Nakai). Food Sci. Biotechnol..

[22] Kwiecień I., Szopa A., Madej K., Ekiert H. (2013). Arbutin Production via Biotransformation of Hydroquinone in In Vitro Cultures of *Aronia melanocarpa* (Michx.) Elliott. Acta Biochim. Pol..

[23] Xu K.-X., Xue M.-G., Li Z., Ye B.-C., Zhang B. (2022). Recent Progress on Feasible Strategies for Arbutin Production. Front. Bioeng. Biotechnol..

[24] Lutterbach R., Stöckigt J. (1992). High-Yield Formation of Arbutin from Hydroquinone by Cell-Suspension Cultures of *Rauwolfia serpentina*. Helv. Chim. Acta.

[25] Inomata S., Yokoyama M., Seto S., Yanagi M. (1991). High-Level Production of Arbutin from Hydroquinone in Suspension Cultures of *Catharanthus roseus* Plant Cells. Appl. Microbiol. Biotechnol..

[26] Casas D.A., Pitta-Alvarez S.I., Giulietti A.M. (1998). Biotransformation of Hydroquinone by Hairy Roots of Brugmansia Candida and Effect of Sugars and Free-Radical Scavengers. Appl. Biochem. Biotechnol..

[27] Kittipongpatana N., Maneerat P., Pattanakitkosol P., Kittipongpatana O. (2007). Effect of Some Factors on the Growth of *Capsicum annuum* L. Cell Suspension Culture and Biotransformation of Hydroquinone to Arbutin. CMU J. Nat. Sci..

[28] Whayne T.F. (2018). Clinical Use of Digitalis: A State of the Art Review. Am. J. Cardiovasc. Drugs.

[29] Coste A., Pop C.E., Butiuc-Keul A., Munteanu-Deliu C., Deliu C., Halmagyi A. (2003). Aspects Concerning Hydroquinone Effect on Cell Cultures of *Arctostaphyllos uva-ursi*, Catharantus Roseus and Digitalis Lanata. Contrib. Bot..

[30] Pop C.E., Deliu C., Tămaș M. (2005). Biotransformarea Hidrochinonei În Arbutozidă În Culturi Celulare In Vitro de Digitalis Lanata. Clujul Med..

[31] Pop C.E., Deliu C., Tămaș M., Coste A. Use of Digitalis Purpurea Cell Cultures for the Biotransformation of Hydroquinone into Arbutin. Proceedings of the 4th Conference on Medicinal and Aromatic Plants of South-East European Countries.

[32] Murashige T., Skoog F. (1962). A Revised Medium for Rapid Growth and Bio Assays with Tobacco Tissue Cultures. Physiol. Plant..

[33] Evans D.E., Coleman J.O.D., Kearns A. (2020). Plant Cell Culture.

[34] Pop C.E., Vlase L., Tămaș M. (2009). Natural Resources Containing Arbutin. Determination of Arbutin in the Leaves of *Bergenia crassifolia* L. Fritsch. Acclimated in Romania. Not. Bot. Horti Agrobot..

[35] Vlase A.-M., Toiu A., Tomuță I., Vlase L., Muntean D., Casian T., Fizeșan I., Nadăș G.C., Novac C.Ș., Tămaș M. (2022). Epilobium Species: From Optimization of the Extraction Process to Evaluation of Biological Properties. Antioxidants.

[36] Solcan M.-B., Fizeșan I., Vlase L., Vlase A.-M., Rusu M.E., Mateș L., Petru A.-E., Creștin I.-V., Tomuțǎ I., Popa D.-S. (2023). Phytochemical Profile and Biological Activities of Extracts Obtained from Young Shoots of Blackcurrant (*Ribes nigrum* L.), European Blueberry (*Vaccinium myrtillus* L.), and Mountain Cranberry (*Vaccinium vitis-idaea* L.). Horticulturae.

[37] Carvalho E.B., Curtis W.R. (1999). The Effect of Inoculum Size on the Growth of Cell and Root Cultures of *Hyoscyamus muticus*: Implications for Reactor Inoculation. Biotechnol. Bioprocess Eng..

[38] Gorret N., Bin Rosli S.K., Oppenheim S.F., Willis L.B., Lessard P.A., Rha C., Sinskey A.J. (2004). Bioreactor Culture of Oil Palm (*Elaeis guineensis*) and Effects of Nitrogen Source, Inoculum Size, and Conditioned Medium on Biomass Production. J. Biotechnol..

[39] Zhang C., Wu J., He G. (2002). Effects of Inoculum Size and Age on Biomass Growth and Paclitaxel Production of Elicitor-Treated Taxus Yunnanensis Cell Cultures. Appl. Microbiol. Biotechnol..

[40] Chan L.K., Lim P.S., Choo M.L., Boey P.L. (2010). Establishment of *Cyperus aromaticus* Cell Suspension Cultures for the Production of Juvenile Hormone III. Vitr. Cell. Dev. Biol. Plant.

[41] Lo K.Y., Nadali B.J., Chan L.-K. (2012). Investigation on the Effect of Subculture Frequency and Inoculum Size on the Artemisinin Content in a Cell Suspension Culture of *Artemisia annua* L. Aust. J. Crop Sci..

[42] Che Saad N., Mazlan F.I., Abd Karim K. (2016). Factors Affecting the Establishment and Growth of Pogostemon Cablin Cell Suspension Cultures. Int. J. Adv. Res. Sci. Eng. Technol..

[43] Sang-Yoon L., Kim D.-I. (2004). Effects of Pluronic F-68 on Cell Growth of Digitalis Lanata in Aqueous Two-Phase Systems. J. Microbiol. Biotechnol..

[44] Tomilova S.V., Kochkin D.V., Tyurina T.M., Glagoleva E.S., Labunskaya E.A., Galishev B.A., Nosov A.M. (2022). Specificity of Growth and Synthesis of Secondary Metabolites in Cultures In Vitro Digitalis Lanata Ehrh. Russ. J. Plant. Physiol..

[45] Dantas L.A., Faria P.S.A., Dário B.M.M., Arantes A.L.M., Silva F.G., Avila R.G., Pereira P.S., Neto A.R. (2021). The Impact of Carbon Source on Cell Growth and the Production of Bioactive Compounds in Cell Suspensions of *Hancornia speciosa* Gomes. Sci. Rep..

[46] See K., Bhatt A., Keng C. (2011). Effect of Sucrose and Methyl Jasmonate on Biomass and Anthocyanin Production in Cell Suspension Culture of *Melastoma malabathricum* (Melastomaceae). Rev. Biol. Trop..

[47] Solís-Ramos L., Carballo L., Valdez-Melara M. (2013). Establishment of Cell Suspension Cultures of Two Costa Rican Jatropha Species Euphorbiaceae. Rev. De Biol. Trop..

[48] Modarres M., Esmaeilzadeh Bahabadi S., Taghavizadeh Yazdi M.E. (2018). Enhanced Production of Phenolic Acids in Cell Suspension Culture of *Salvia leriifolia* Benth. Using Growth Regulators and Sucrose. Cytotechnology.

[49] Yokoyama M., Inomata S., Seto S., Yanagi M. (1990). Effects of Sugars on the Glucosylation of Exogenous Hydroquinone by *Catharanthus roseus* Cells in Suspension Culture. Plant Cell Physiol..

[50] Yokoyama M., Inomata S., Yanagi M., Wachi Y. (1996). Change of Maximal Cellular Productivity of Arbutin by Biotransformation Depending on the Culture Stage of *Catharanthus roseus* Cells. Plant Tissue Cult. Lett..

[51] Suzuki T., Yoshioka T., Tabata M., Fujita Y. (1987). Potential of *Datura innoxia* Cell Suspension Cultures for Glucosylating Hydroquinone. Plant Cell Rep..

[52] Tofighi Z., Amini M., Shirzadi M., Mirhabibi H., Ghazi Saeedi N., Yassa N. (2016). Vigna Radiata as a New Source for Biotransformation of Hydroquinone to Arbutin. Pharm. Sci..

[53] Kumar P.P., Joy R.W., Thorpe T.A. (1990). Ethylene and Carbon Dioxide Accumulation, and Growth of Cell Suspension Cultures of *Picea glauca* (White Spruce). J. Plant Physiol..

[54] Akalezi C.O., Liu S., Li Q.S., Yu J.T., Zhong J.J. (1999). Combined Effects of Initial Sucrose Concentration and Inoculum Size on Cell Growth and Ginseng Saponin Production by Suspension Cultures of Panax Ginseng. Process Biochem..

[55] Singh M., Chaturvedi R. (2012). Evaluation of Nutrient Uptake and Physical Parameters on Cell Biomass Growth and Production of Spilanthol in Suspension Cultures of *Spilanthes acmella* Murr. Bioprocess Biosyst. Eng..

[56] Jayaraman S., Daud N.H., Halis R., Mohamed R. (2014). Effects of Plant Growth Regulators, Carbon Sources and pH Values on Callus Induction in *Aquilaria malaccensis* Leaf Explants and Characteristics of the Resultant Calli. J. For. Res..

[57] Ali M., Abbasi B.H., Ahmad N., Ali S.S., Ali S., Ali G.S. (2016). Sucrose-Enhanced Biosynthesis of Medicinally Important Antioxidant Secondary Metabolites in Cell Suspension Cultures of *Artemisia absinthium* L. Bioprocess Biosyst. Eng..

[58] Kittipongpatana N., Chaiwan A., Pusod U., Kittipongpatana O.S. (2007). High-Performance Liquid Chromatographic Method for Separation and Quantitative Analysis of Arbutin in Plant Tissue Cultures. CMU J. Nat. Sci..

[59] Duskova J., Dusek J., Jahodar L., Poustka F. (2005). Arbutin, Salicin: The Possibilities of Their Biotechnological Production. Ceska Slov. Farm..

[60] Yan C.-Y., Zhang Z., Yu R.-M., Kong L.-Y. (2007). Studies on Biotransformation of Arbutin by 4-Hydroxy Phenol in Hairy Root of Polygonum Multiflorum. Zhongguo Zhong Yao Za Zhi.

